# Supplementary material for: Multi‐dataset identification of innovative feature genes and molecular mechanisms in keratoconus
Source: J Cell Mol Med. 2024 Sep 19;28(18):e70079. doi: 10.1111/jcmm.70079 (PMC11412914; doi:10.1111/jcmm.70079)
Supplement: Supplementary file 2 — Material S2. [file JCMM-28-e70079-s002.zip › 7.GSEA analysis/2.ATOH7/KEGG_ASCORBATE_AND_ALDARATE_METABOLISM.html]

Details for gene set KEGG\_ASCORBATE\_AND\_ALDARATE\_METABOLISM[GSEA]

|  || Dataset | ATOH7.group.cls#h\_versus\_l |
| Phenotype | group.cls#h\_versus\_l |
| Upregulated in class | l |
| GeneSet | KEGG\_ASCORBATE\_AND\_ALDARATE\_METABOLISM |
| Enrichment Score (ES) | -0.36343613 |
| Normalized Enrichment Score (NES) | -0.993619 |
| Nominal p-value | 0.44510978 |
| FDR q-value | 1.0 |
| FWER p-Value | 1.0 |
Table: GSEA Results Summary

  

Fig 1: Enrichment plot: KEGG\_ASCORBATE\_AND\_ALDARATE\_METABOLISM      
 Profile of the Running ES Score & Positions of GeneSet Members on the Rank Ordered List

  

| SYMBOL | TITLE | RANK IN GENE LIST | RANK METRIC SCORE | RUNNING ES | CORE ENRICHMENT || 1 | ALDH2 | na | 221 | 0.659 | 0.1887 | No |
| 2 | UGT2B7 | na | 1726 | 0.367 | 0.2539 | No |
| 3 | UGT2B15 | na | 6980 | 0.144 | 0.1450 | No |
| 4 | ALDH9A1 | na | 7741 | 0.125 | 0.1600 | No |
| 5 | ALDH1B1 | na | 7850 | 0.122 | 0.1929 | No |
| 6 | MIOX | na | 8558 | 0.107 | 0.2043 | No |
| 7 | UGT2A1 | na | 8781 | 0.103 | 0.2283 | No |
| 8 | UGDH | na | 12373 | 0.048 | 0.1389 | No |
| 9 | UGT2B11 | na | 13663 | 0.034 | 0.1118 | No |
| 10 | ALDH7A1 | na | 14436 | 0.026 | 0.0973 | No |
| 11 | UGT2A3 | na | 16675 | 0.007 | 0.0350 | No |
| 12 | UGT2B10 | na | 19029 | -0.013 | -0.0289 | No |
| 13 | ALDH3A2 | na | 20819 | -0.030 | -0.0716 | No |
| 14 | UGT2B4 | na | 30951 | -0.245 | -0.2909 | Yes |
| 15 | UGT1A6 | na | 33234 | -0.388 | -0.2420 | Yes |
| 16 | UGT2B28 | na | 33254 | -0.390 | -0.1271 | Yes |
| 17 | UGT1A8 | na | 34302 | -0.573 | 0.0123 | Yes |
Table: GSEA details [plain text format]

  

Fig 2: KEGG\_ASCORBATE\_AND\_ALDARATE\_METABOLISM      
 Blue-Pink O' Gram in the Space of the Analyzed GeneSet

  

Fig 3: KEGG\_ASCORBATE\_AND\_ALDARATE\_METABOLISM: Random ES distribution      
 Gene set null distribution of ES for **KEGG\_ASCORBATE\_AND\_ALDARATE\_METABOLISM**
